# Management of an Acute Thermal Injury With Subatmospheric Pressure

**Published:** 2005-03-24

**Authors:** Joseph A. Molnar, Jordan L. Simpson, Denise M. Voignier, Michael J. Morykwas, Louis C. Argenta

**Affiliations:** Department of Plastic and Reconstructive Surgery, Wake Forest University School of Medicine, Winston-Salem, NC

## Abstract

**Objective:** This article reports the first application of subatmospheric pressure management to a deep, partial-thickness human thermal burn. **Methods:** After cleaning the wound, the decision was made to treat the hand and distal forearm with subatmospheric pressure (V.A.C., KCI, Inc, San Antonio, Tex). The sponge was applied directly to the burned skin without additional interface at approximately 6 hours after injury. The dressing was maintained at a continuous negative pressure of 125 mm Hg over the next 40 hours, with interruption only for routine clinical evaluation at 5, 16, and 24 hours after initiation of treatment. This was accomplished by opening the dressing without completely changing it. The treatment was tolerated well by the patient, requiring no excessive pain medication. After the subatmospheric pressure treatment was stopped, the wound appeared to be of indeterminate depth and the patient was started on twice daily applications of silver sulfadiazine. **Results:** The clinical impression at this time was that the hand burn had not progressed but had stabilized and had minimal edema. He was followed as an outpatient and returned to work by 8 weeks. At approximately 4 weeks postinjury, his skin not only was functional but also appeared more normal, with less hyperemia than adjacent areas treated with topical antibacterials. **Conclusion:** The present case does not prove that subatmospheric pressure treatment prevents burn wound progression. However, when combined with the previously reported laboratory studies it suggests the need for further research. Currently, a prospective, randomized, blinded, controlled multicenter trial is underway to evaluate the clinical importance of these observations.

Management of acute thermal injury is often frustrated by the phenomenon of burn wound progression. In this circumstance, heat-damaged tissue that is alive at presentation becomes progressively nonviable until the skin is found to be nonsalvageable and requires excision and grafting. The etiology of this process is unclear. It has been best described by Jackson as a zone of stasis where with increased vascular permeability, progressive edema, increased blood viscosity, and vascular thrombosis the tissue dies.^1^ While limiting burn wound progression would be of clear benefit to the burn patient, no clinical studies have shown a way to prevent it.^2^

The V.A.C. (K.C.I., Inc, San Antonio, Tex) consists of an open cell polyurethane ether foam with an embedded evacuation tube. The foam is sealed to the wound with an adherent drape, and subatmospheric pressure is applied to the evacuation tube. Previous studies have demonstrated the effectiveness of this device in helping to control edema and speed up the vascularization of wounds.^3^ Morykwas et al have also demonstrated in a swine model of thermal injury that the maximum depth of cell death could be decreased with application of subatmospheric pressure.^4^

We report the first application of subatmospheric pressure management to a deep, partial-thickness human thermal burn.

## CASE REPORT

**Figure 1:**
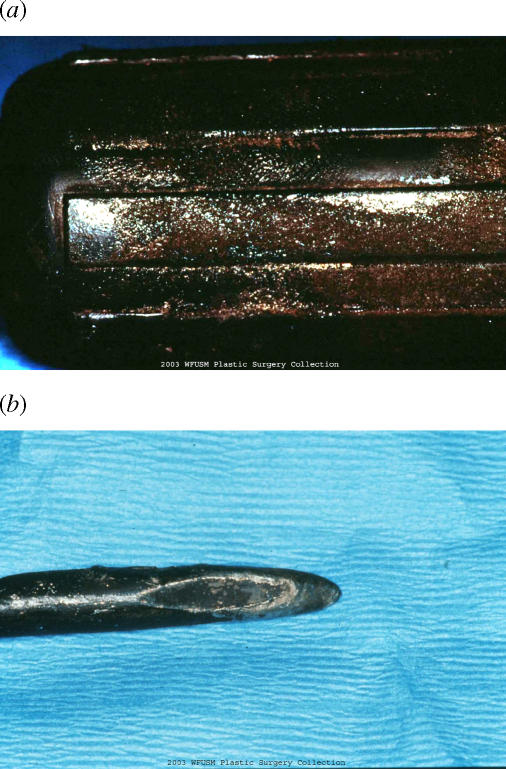
Handle of screwdriver (a) and screwdriver tip (b) used by electrician described in text. Note that fingerprint melted into the handle and that the tip of the screwdriver has been rounded.

On August 2, 1995, a 26-year-old male electrician received a flash burn to his right upper extremity and face when exposed to the heat from a high-voltage electrical arc (Fig 1).

**Figure 2:**
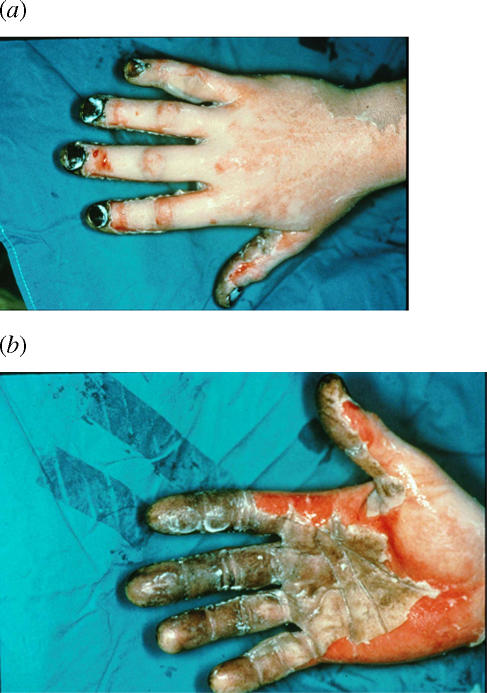
Right hand (dorsum [a]; palmar aspect [b]) of patient at time of arrival. Outside referral center had treated the hand with silver sulfadiazine before transfer.

The hand and digits were pale in color and dry on the dorsum, suggesting deep, partial-thickness burns (Fig 2). The injury was progressively more superficial proximally on the extremity. The clinical impression of 3 surgeons with experience in burn care was that the distal portion of the burn, ie, hand and forearm, would require excision and grafting.

After cleaning the wound, the decision was made to treat the hand and distal forearm with subatmospheric pressure (V.A.C., KCI, Inc, San Antonio, Tex) and to apply silver sulfadiazine more proximally. The sponge was applied directly to the burned skin without additional interface at approximately 6 hours after injury. The dressing was maintained at a continuous negative pressure of 125 mm Hg over the next 40 hours, with interruption only for routine clinical evaluation at 5, 16, and 24 hours after initiation of treatment. This was accomplished by opening the dressing without completely changing it. The treatment was tolerated well by the patient, requiring no excessive pain medication. After the subatmospheric pressure treatment was stopped, the patient was started on twice daily applications of silver sulfadiazine.

**Figure 3:**
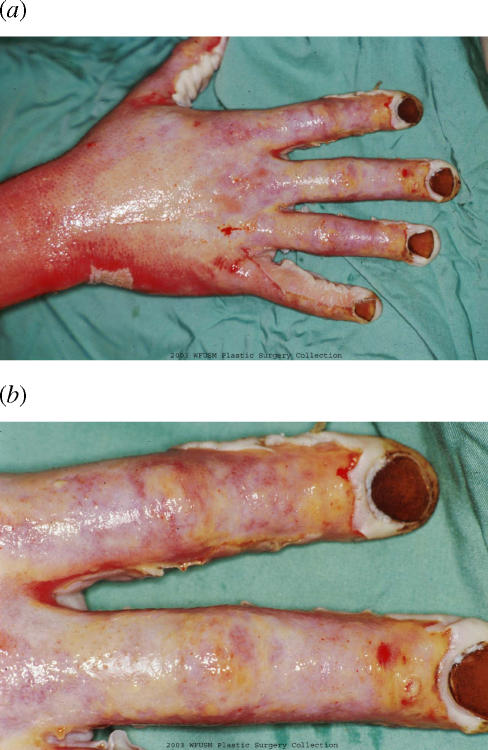
Right hand after 48 hours of subatmospheric pressure treatment (a). Close-up of digits (b).

The clinical impression at this time was that the hand burn had not progressed but had stabilized and had minimal edema (Fig 3). However, it was now of indeterminate depth.

The patient was started on hand therapy, and the hand was kept elevated. The wound continued to epithelialize until it was clinically healed by Day 10, but the patient had received significant fingernail injury that persisted until the nail was completely replaced.

**Figure 4:**
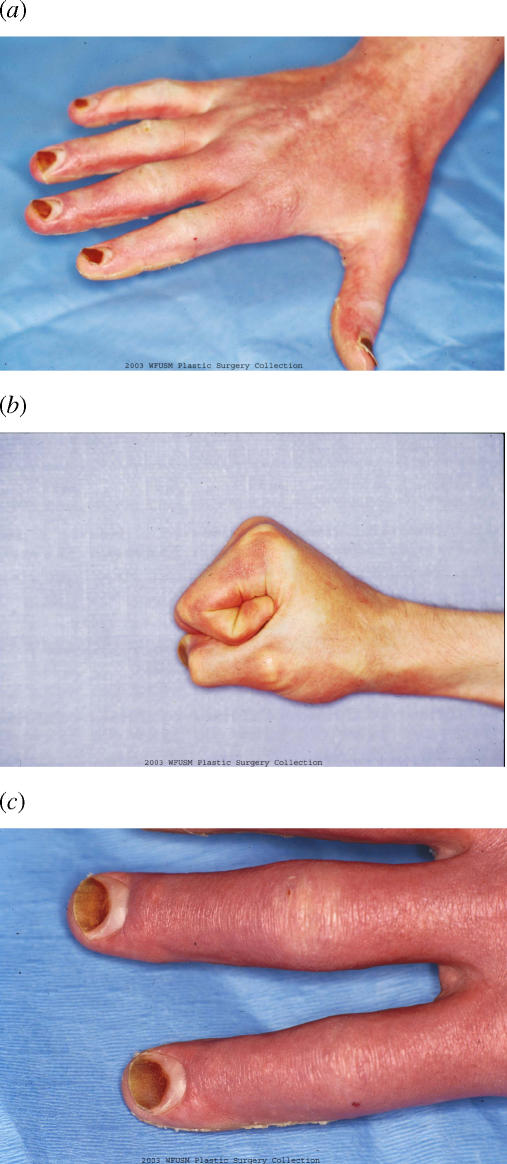
Right hand at approximately 5 weeks after injury. Digits in extension (a). Digits in flexion (b) and close-up of digits (c). Note the severity of injury to the fingernails, suggesting a deep burn to the hand.

**Figure 5:**
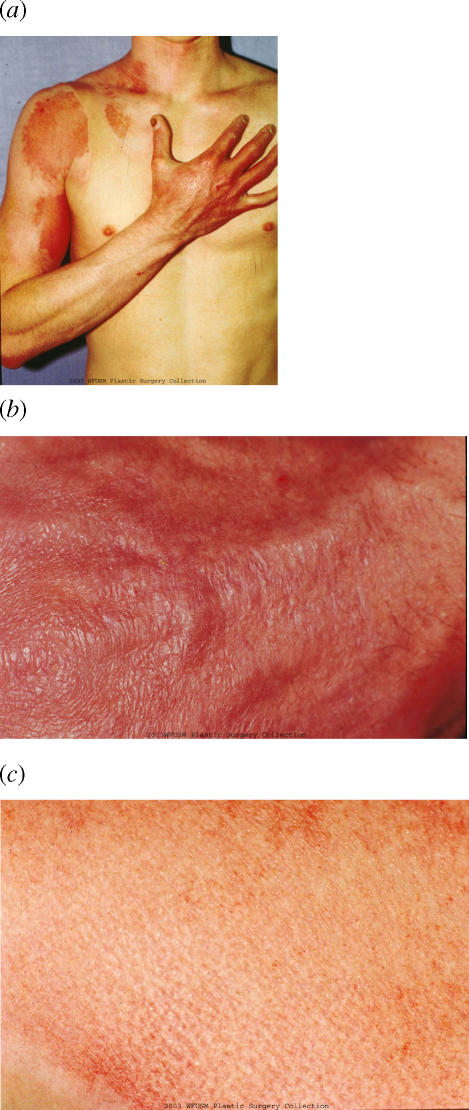
Appearance of the upper extremity at approximately 5 weeks (a). Close-up of the skin of the dorsum of the hand (b) as compared to a close-up of the skin of the shoulder (c). Note that the skin of the shoulder, which appeared more superficially burned than the hand on arrival, now has more discoloration than the skin of the hand.

The patient was discharged home on the 12th postinjury day. He was followed as an outpatient and returned to work by October. At approximately 4 weeks, his skin not only was functional but also had an excellent cosmetic result (Fig 4). In addition, the skin on the hand appeared more normal with less hyperemia than the skin of the shoulder, despite the fact that the hand had received the deepest burn consistent with the mechanism of surgery (Fig 5).

## DISCUSSION

Fifty years ago, Jackson put forth a paradigm for an understanding of the pathogenesis of burn wound progression.^1^ He described the wound as consisting of 3 concentric zones of injury. The most severe of these is the zone of coagulation. It is irreversibly damaged and represents nonsalvageable dead tissue. To the other extreme is the zone of hyperemia. This tissue is minimally injured, resulting in an inflammatory response, and will usually heal spontaneously. In between is the zone of stasis. This is characterized by increased vascular permeability, edema, and progressive blood viscosity, leading to thrombosis and additional tissue death. It is this zone of stasis that represents the deep second-degree burn that is clearly viable tissue when the patient arrives but subsequently goes on to die and requires excision and grafting much in the manner of a third-degree or full-thickness burn.

While initially Jackson thought that such capillary stasis and burn wound progression was an inevitable consequence of the original injury, Order et al^5^ demonstrated reopening of the circulation in second-degree burns in a rat model more than a decade later. This led Jackson and others to consider the possibility of prohibiting the progression so as to minimize the potential need for surgery and perhaps to save lives.^6^ However, in order to control this process, it would be necessary to understand the mechanism.

Evaluation of the microcirculatory changes due to thermal injury has demonstrated the complex nature of the response. Early after the injury, endothelial cells swell, resulting in capillary narrowing and decreased flow.^7^ The swelling of the endothelial cells contributes to capillary leak but may also be the result of free-radical mechanisms.^2^,^8^,^9^ The capillary leak allows margination of cellular elements of the blood, platelet aggregation, and stimulation of inflammatory mediator response. This process begins in the first 3 to 24 hours depending on the severity of injury and continues for up to 48 hours after burn.^4^,^5^,^9^ While much of the emphasis of microcirculatory clotting has been on the arteriole, it appears that venous occlusion may occur first, resulting in secondary arteriolar clotting.^10^–^13^

In addition to inflammation and progressive thrombosis, more direct mechanisms may cause progressive tissue damage. Zawacki and others have shown that dehydration due to the loss of the outer protective layers may contribute to burn wound progression.^14^–^16^ Systemic hypoperfusion, infection, malnutrition, and inadequate immune response are all important causes of worsening of the burn wound, and proper resuscitation and metabolic support limit this process.^13^,^14^

Efforts to limit burn wound progression have primarily concentrated on pharmacologic interventions in the thrombosis or inflammatory response. Robson et al showed that application of 1% methylprednisolone acetate to a guinea pig model of burn wounds decreased loss of dermal appendages and increased dermal perfusion, presumably by interfering with white blood cell adherence.^17^ However, this was not confirmed by subsequent investigators using clobestasol propionate.^18^ Use of monoclonal antibodies to prevent leukocyte adherence in a burn model did decrease burn size, speed up reepithelialization, produce thinner eschar, spare more hair follicles, and have greater patency of vessels than controls.^19^,^20^ While Ehrlich found that a lazaroid could prevent burn wound progression, Melikian et al could not find an effect of the free-radical mechanism on burn wound progression using dimethyl sulfoxide, allopurinol, or polyethylene glycol-superoxide dismutase (PEG-SOD; a superoxide scavenger).^9^,^21^

Ehrlich has also demonstrated the importance of the clotting mechanism in this process by the use of ancrod, a protease derived from pit vipers that converts fibrinogen to a nonclotting molecule.^22^ By giving this to rats 3 days before creating experimental burns, he was able to limit the size of the burn. Heparin has also been used anecdotally to treat clinical burns by preventing worsening by thrombosis.^23^ However, the most consistent effect has been seen with the use of the nonsteroidal anti-inflammatory drug ibuprofen. While early reports suggested a thromboxane mechanism to prevent burn wound progression, more recent studies have suggested that it works by blocking a plasmin inhibitor that would normally block fibrinolysis in the burn wound.^2^,^24^–^26^ Finally, a topical form of ibuprofen (flurbiprofen) when applied to an acute burn model within 4 hours of injury has also been suggested to have a positive effect on vascularity.

Despite these efforts, no definitive technique has been shown to be clinically effective in minimizing burn wound progression. This is because the pharmacologic methods described are either toxic or contraindicated or must be administered before or too early after injury to be effective. Since the average burn patient arrives at the hospital 3 hours after injury,^27^ therapeutic interventions must take this into consideration. The ideal technique to stop burn wound progression would allow for a 3 or more hour delay before hospital treatment, would have no systemic effects, and would not interfere with other treatment methods.

Morykwas et al evaluated the effect of subatmospheric pressure on acute burn wounds in a swine model.^4^ He applied a relative negative pressure of 125 mm Hg in an artificially closed space to experimental wounds with control wounds on the same animal. When applied within 12 hours after injury, a significant improvement was found as measured by the maximum depth of cell death. Based on this evaluation, treatment periods as short as 6 hours were efficacious. In fact, application periods as long as 5 days were not significantly different from application periods as short as 6 or 12 hours. In addition, histologic evaluation demonstrated decreased inflammatory response in wounds treated with subatmospheric pressure as compared to controls.

In the present study, subatmospheric pressure treatment was applied to an upper extremity in a patient with a flash burn that extended from his fingertips to over his shoulder. This is the first clinical application of subatmospheric pressure to an acute human burn injury. The treatment period was approximately 2 days, and it was applied approximately 6 hours after injury. Despite the clinical impression of 3 surgeons experienced in burn care that this would ultimately require excision and grafting, this was avoided. The wound healed without complication, and applying subatmospheric pressure to the acutely burned tissue did no harm. In addition, the skin that initially appeared the deepest burned on the hand and the forearm healed in a manner that was less hyperemic with superior skin quality to the skin of the shoulder that received less injury.

Unfortunately, there is no absolute method of evaluation that the burn surgeon may use to ascertain the depth of the burn at this early time point. Recent reports using scanning laser Doppler have had some interest but are not without error and not widely used to make such decisions.^28^ The evaluation of burn depth remains primarily a clinical decision. It is impossible to know with certainty if this patient would have healed as well with alternative treatments such as silver sulfadiazine. Nonetheless, the severe nail damage, as seen in Figure 4, suggests that excision and grafting would have been necessary.

A more intriguing issue is how subatmospheric pressure treatment improves the healing of the burn wound as seen in the laboratory studies of Morykwas et al^4^ and as suggested by this clinical case. One possible way is that the device removes acute inflammatory mediators, such as free radicals and cytokines, that are involved with burn wound progression.^2^,^8^,^9^,^20^,^21^ While this has not been proven in burns, it is clear from the studies in crush injury that certain toxins may be removed from acute wounds.^29^ It is also possible that decreasing edema is an important mechanism to speed up the healing of the acute burn. With edema there is a decrease in vascular density, increased diffusion distance, possible vasospasm, thrombosis, and stasis in the microcirculation. Present observations suggest that subatmospheric pressure treatment does decrease wound edema by yet uncertain mechanisms. Finally, subatmospheric pressure treatment may provide an ideal environment for the healing wound by providing the damaged skin with the ideal water vapor pressure to avoid desiccation.^14^–^16^

The present case does not prove that subatmospheric pressure treatment prevents burn wound progression. However, when combined with the previously reported laboratory studies it suggests the need for further research. Currently, a prospective, randomized, blinded, controlled multicenter trial is underway to evaluate the clinical importance of these observations.
